# Clinical training under movement restriction: checkpoint burden, burnout, and intentions to train abroad among medical students in Palestine

**DOI:** 10.1186/s12909-026-09049-2

**Published:** 2026-03-21

**Authors:** Nouraldeen Deeb, Salahaldeen Deeb, Alhareth M. Amro, Khaled Alhashlamon, Ammir Abuzahra, Samia hamad, Farid K. Abu shama, Abdallah Qawasmeh

**Affiliations:** 1 Faculty of Medicine, Al-Quds University, Jerusalem, Palestine; 2 Faculty of Medicine, Hebron University, Jerusalem, Palestine; 3 Faculty of Medicine, An-Najah University, Jerusalem, MD Palestine; 4 Health Education and Scientific Research Unit, Minstry of Health, Ramallah, Palestine

**Keywords:** Palestinian cities in the West Bank, Military checkpoint, Burnout, Medical Doctors, Restricted road

## Abstract

**Background:**

Medical education in Palestine occurs amid checkpoint-related movement restrictions that may affect well-being and career plans. We assessed burnout, perceived clinical training adequacy, checkpoint burden, and intention to pursue residency abroad among clinical-year medical students.

**Methods:**

We conducted a cross-sectional online survey in 2025 among 4th–6th year medical students from five West Bank medical schools (*N* = 371). Burnout and perceived training adequacy were assessed using study-specific multi-item Likert composites adapted from previously published instruments and piloted in Arabic for this context. Checkpoint burden was summarized as a standardized index derived from checkpoint-crossing frequency, delayed arrival or missed training time, and missed clinical days. Intention to pursue residency training abroad was coded as yes versus no/unsure.

**Results:**

Mean burnout was 3.29 ± 0.83 and perceived training adequacy was 3.18 ± 0.70; 59.3% of respondents intended to pursue residency training abroad. Burnout was negatively correlated with perceived training adequacy (*r* = − 0.276, *p* < 0.0001) and positively correlated with checkpoint burden (*r* = + 0.245, *p* < 0.00001), whereas perceived training adequacy was not associated with checkpoint burden (*r *= + 0.022, *p* = 0.668). In multivariable logistic regression, higher burnout independently predicted intention to pursue residency training abroad (OR 1.57, 95% CI 1.19–2.08; *p* = 0.0015), and male sex was associated with higher odds (OR 2.18, 95% CI 1.39–3.42; *p* = 0.0007), whereas higher checkpoint burden (OR 0.68, 95% CI 0.48–0.96; *p* = 0.029) and East Jerusalem residence (OR 0.26, 95% CI 0.13–0.49; *p* < 0.0001) were associated with lower odds of intending to train abroad.

**Conclusions:**

Higher burnout was associated with greater checkpoint burden and with intention to pursue residency training abroad. Perceived training adequacy was not associated with checkpoint burden. Given the cross-sectional design and the use of adapted, not formally validated, composite measures, these findings should be interpreted cautiously; nevertheless, they highlight the need for student support and for measures that reduce the educational impact of mobility restrictions.

## Introduction

Burnout is a work-related syndrome that develops in response to chronic, unmanaged stress and is commonly conceptualized in healthcare training as a triad of emotional exhaustion, depersonalization/cynicism, and reduced professional efficacy [1–3]. Among medical trainees, burnout is associated with adverse mental health outcomes, impaired learning and performance, and downstream risks for workforce stability and patient safety [4, 5]. 

The clinical phase of medical education is a particularly high-risk period, as students face heavier workload, frequent evaluations, emotionally demanding encounters, and variable quality of supervision and feedback [6, 7]. In resource-constrained settings, these stressors may have outsized effects because clinical teaching infrastructure and staffing are often limited and unevenly distributed [7]. 

In conflict-affected environments, standard academic demands interact with structural constraints that disrupt training continuity and increase psychological strain [8]. Barriers to reaching clinical sites such as security checkpoints, road closures, and unpredictable travel delays can reduce clinical exposure and amplify daily stress, potentially contributing to burnout and shaping career plans [8]. 

The occupied Palestinian territory, particularly the West Bank, represents a setting where movement restriction is a persistent feature of daily life and health-system function [9, 10]. Medical students may be required to cross military checkpoints to reach training hospitals, often encountering unpredictable delays, increased cost, and missed clinical time [10, 11]. These conditions may generate a measurable “checkpoint burden” and could plausibly influence both trainee well-being (burnout) and future workforce intentions, including interest in residency training abroad a phenomenon closely linked to broader concerns about professional emigration (“brain drain”) from Palestine [9, 12]. However, quantitative evidence simultaneously linking checkpoint burden, burnout, and intentions to train abroad among Palestinian medical students remains limited. Therefore, this study assesses checkpoint burden and burnout among clinical-year medical students in the West Bank and examines their associations with perceived clinical training adequacy and intention to pursue residency training abroad.

## Methodology

### Study design and setting

A multi-centre cross-sectional online survey was conducted between January and April 2025 to examine how checkpoint-related movement restrictions relate to burnout, perceived clinical training adequacy, and intentions to pursue residency training abroad among medical students in the West Bank. The questionnaire was administered via Google Forms. Because clinical rotations are distributed across multiple geographically separated hospitals, an online format maximised accessibility and allowed participation without disrupting clinical duties. Recruitment occurred through university networks and social media using convenience and snowball sampling, which supported broad dissemination but reduced researcher control over sample diversity.

### Participants

Eligible participants were clinical-year medical students (4th–6th year) enrolled at Al-Quds University, An-Najah National University, Hebron University, Palestine Polytechnic University, or the Arab American University of Palestine; pre-clinical students were excluded because they do not routinely undertake hospital-based training. There were no restrictions by gender, region of residence, or commuting pattern. Participation was voluntary and anonymous, and access was conditional on electronic informed consent. A priori, sample size was estimated for a cross-sectional survey using the single-proportion formula, n = Z²p(1 − p)/d². Assuming 50% prevalence, 95% confidence, and 6% precision, the minimum required sample was 267; after inflating by 10% for incomplete responses, the target sample was approximately 300 students. A total of 371 students completed the survey.

### Questionnaire development and translation

The questionnaire was a study-specific instrument developed for this project using items adapted from previously published studies on burnout, training adequacy, and mobility-related educational barriers [13–15]. We did not administer a single established burnout or training-adequacy instrument in its original validated form; instead, we selected and adapted relevant items to reflect the Palestinian clinical-training context, including checkpoint-related disruption. The draft questionnaire was forward-translated into Arabic, reviewed for clarity and contextual relevance, and piloted with 30 clinical-year students. Feedback from the pilot was used to refine wording, examples, and skip logic. Additional locally developed items on checkpoint-crossing frequency, travel delays, and missed clinical days were informed by student focus-group input. The final survey covered demographics, mobility barriers, burnout-related experiences, perceived clinical training adequacy, and intention to pursue residency training abroad. Because the final adapted instrument was not formally psychometrically validated, the present study reports internal-consistency estimates and treats the scale scores as exploratory composite measures.

### Scoring and reliability

The burnout and training-adequacy scores were treated as study-specific composite measures rather than previously validated scales administered unchanged. Reliability was assessed using Cronbach’s α, and construct validity was explored indirectly by examining whether associations between checkpoint burden, burnout, and training adequacy followed the hypothesized direction.

Burnout was assessed using a study-specific seven-item composite informed by prior burnout literature and adapted for the local clinical-training context [1–3]. Burnout was operationalized as the mean of seven 5-point Likert items (1 = strongly disagree to 5 = strongly agree): (1) “I feel emotionally drained by my clinical training,” (2) “I have become less enthusiastic about my medical education,” (3) “I rarely feel that my work as a student doctor makes a difference,” (4) “I feel burned out by my studies,” (5) “I doubt the value of what I’m learning in my clinical years,” (6) “Stress related to reaching clinical sites (e.g., checkpoints, travel) contributes to emotional exhaustion,” and (7) “I struggle to recover my energy between clinical days.” Higher scores indicated greater burnout. Internal consistency was high in this sample (Cronbach’s α = 0.85; complete-case *n* = 370).

Perceived clinical training adequacy was assessed using a study-specific seven-item composite adapted from published work on clinical supervision, confidence, and preparedness for practice. confidence performing basic clinical tasks, confidence presenting patients on rounds, perceived preparedness for future practice, adequacy of supervision/feedback, supervisor availability, adequacy of opportunities to examine patients/perform appropriate tasks, and whether training hospitals were well equipped for medical education. Higher scores indicated better perceived training adequacy. Internal consistency was high (Cronbach’s α = 0.83; complete-case *n* = 369).

Checkpoint burden was operationalized as a composite standardized index derived from three ordinal mobility-exposure measures: checkpoint-crossing frequency (Never/Rarely/For some rotations/For nearly all or all rotations), frequency of arriving late or missing part of a clinical day due to access barriers (Never/1–2 times/Monthly/Weekly/Almost daily), and having missed an entire clinical day due to access barriers (No/Yes once or twice/Yes more than twice). Each component was coded in ascending order of exposure, standardized (z-scores), and averaged; higher values indicated greater checkpoint burden. As an exploratory check of construct validity, we examined whether higher checkpoint burden was associated with higher burnout and whether higher burnout was associated with lower perceived training adequacy, as hypothesized a priori; these analyses do not constitute full psychometric validation.

## Data management and statistical analysis

Descriptive statistics summarized participant characteristics and key exposures/outcomes. Group comparisons used t-tests or ANOVA for continuous outcomes and χ² tests for categorical outcomes. Pearson correlations assessed relationships between continuous composite measures.

Multivariable logistic regression was used to estimate adjusted odds ratios (ORs) for intention to pursue residency training abroad (Yes vs. No/Unsure). To ensure interpretability and avoid overfitting, the final model included the study’s pre-specified exposure variables and key sociodemographic covariates: checkpoint burden (standardized; OR per 1 SD increase), burnout score (OR per 1-point increase), sex (male vs. female), and residence in East Jerusalem (vs. other regions). Variables were entered simultaneously. Analyses used complete-case data for variables included in each model, and statistical significance was set at *p* < 0.05.

### Ethical approval

Ethical approval was obtained from the Institutional Review Board Committee of Al-Quds University, Jerusalem, Palestine (42/REC/2025). Electronic informed consent was obtained from all participants before they accessed the questionnaire. No identifying information was collected, participation was voluntary, and responses were analysed anonymously and confidentially.

## Results

A total of 371 medical students (206 women, 165 men) completed the survey (Table 1). The sample was predominantly aged 21–22 years (57.4%), with 23–24-year-olds comprising 37.2% and only 5.4% aged ≤ 20 or ≥ 25 years. Participants were in the 4th (23.2%), 5th (42.0%) or 6th (34.8%) clinical year. All five West Bank universities were represented, most commonly Al-Quds University (31.0%), followed by An-Najah (22.1%). Most students resided in the south West Bank (Bethlehem/Hebron, 52.3%), with fewer in northern (24.0%). The distributions of commute time and cost were broad (Table 1). Cronbach’s α for the burnout and training adequacy scales were 0.85 and 0.83, respectively, indicating high internal consistency. The mean burnout score was moderate (3.29 ± 0.83 on a 1–5 scale), while perceived clinical training adequacy was also moderate (3.18 ± 0.70). Overall, 59.3% of respondents intended to pursue residency training abroad (Yes = 220; No/Unsure = 151, comprising No = 40 and Unsure = 111).

**Table 1:** Demographic characteristics of the clinical-year medical students surveyed (*N* = 371)

Characteristic	Category	*n* (%)
**Age (years)**	20 or younger	12 (3.2)
	21–22	213 (57.4)
	23–24	138 (37.2)
	≥ 25	8 (2.2)
**Gender**	Female	206 (55.5)
	Male	165 (44.5)
**Year of Study**	4th year	86 (23.2)
	5th year	156 (42.0)
	6th year	129 (34.8)
**Region of Residence**	South West Bank (Bethlehem, Hebron)	194 (52.3)
	North West Bank (Jenin, Nablus, etc.)	89 (24.0)
	East Jerusalem	51 (13.8)
	Central West Bank (Ramallah, Jericho)	34 (9.2)
	1948 Territories (Nazareth etc.)	18 (5.0)
**University**	Al-Quds University	115 (31.0)
	An-Najah National University	82 (22.1)
	Hebron University	85 (22.9)
	Palestine Polytechnic University	65 (17.5)
	AAUP (Arab Am. Univ.)	24 (6.5)
**Median commute time**	≤ 30 min	59 (15.9)
	30–60 min	136 (36.6)
	1–2 h	126 (34.0)
	> 2 h	9 (2.4)
	Varies by rotation	41 (11.1)
**Median commute cost per day (NIS)**	≤ 10	12 (3.2)
	10–20	69 (18.6)
	20–30	129 (34.8)
	> 30	113 (30.5)
	Varies by rotation	48 (12.9)
**Crossing military checkpoints**	Never	16 (4.3)
	Rarely	39 (10.5)
	Some rotations	175 (47.2)
	Nearly all rotations	141 (38.0)
**Late/missed clinical days**	Never	37 (10.0)
(due to checkpoint delays)	1–2 times/yr	131 (35.3)
	Monthly	89 (24.0)
	Weekly	84 (22.6)
	Almost daily	30 (8.1)
**Ever missed entire clinical day**	No, never	138 (37.2)
	Yes, once or twice	172 (46.4)
	Yes, > 2 times	61 (16.4)
**Primary training hospital affects**	Checkpoints only	247 (66.6)
	Both checkpoints and distance	30 (8.1)
	Missing/other	94 (25.3)

Group comparisons (Table 2) revealed that burnout scores varied by residence region (ANOVA F(4, 366) = 4.81, *p* = 0.0009). Students from the South West Bank (Bethlehem/Hebron) had the lowest mean burnout (3.12 ± 0.86) compared to those in East Jerusalem (3.61 ± 0.84) and the 1948 territories (3.57 ± 0.80); post-hoc tests showed the South West Bank mean was significantly lower than East Jerusalem (*P* = 0.0004) and the 1948 territories (*P* = 0.036) and also lower than the Central West Bank (Ramallah/Jericho, *P *= 0.031). By contrast, training adequacy scores did not differ significantly across regions (*p* = 0.121). Burnout did not differ by gender (women 3.35 ± 0.85 vs. men 3.22 ± 0.82; t(369) = –1.49, *p* = 0.136) or by year of study (*p *= 0.887). Training adequacy showed a small borderline difference by gender, with men reporting slightly higher scores than women (3.28 ± 0.73 vs. 3.14 ± 0.70; t(369) = 2.00, *p* = 0.046), whereas no difference was observed by year of study (*p* = 0.823).

**Table 2:** Burnout and training adequacy scores by demographic group. Means ± SD are shown. *P*-values are for overall group comparisons (ANOVA or *t*-test) within each factor

Factor	Category	*N*	Burnout (mean ± SD)	Training (mean ± SD)	*p* (burnout)	*p* (training)
**Gender**	Female	206	3.35 ± 0.85	3.14 ± 0.70	0.136	0.046
	Male	165	3.22 ± 0.82	3.28 ± 0.73		
**Age (years)**	≤ 20	12	2.94 ± 1.02	3.05 ± 0.83	0.074	0.717
	21–22	213	3.28 ± 0.82	3.19 ± 0.70		
	23–24	138	3.31 ± 0.80	3.24 ± 0.67		
	≥ 25	8	3.92 ± 1.12	3.09 ± 0.62		
**Region**	South West Bank (Bethlehem/Hebron)	194	3.12 ± 0.86	3.24 ± 0.70	0.0009	0.121
	North West Bank (Jenin/Nablus/Tulkarm)	89	3.33 ± 0.72	3.28 ± 0.61		
	East Jerusalem	51	3.61 ± 0.84	2.98 ± 0.80		
	Central West Bank (Ramallah/Jericho)	34	3.47 ± 0.81	3.18 ± 0.69		
	1948 Territories (Nazareth/Umm al-Fahm)	18	3.57 ± 0.80	3.08 ± 0.63		
**University**	Al-Quds University	115	3.55 ± 0.84	2.99 ± 0.72	< 0.001	0.0013
	An-Najah Nat’l University	82	3.37 ± 0.71	3.30 ± 0.67		
	AAUP (Palestine)	24	3.15 ± 0.79	3.42 ± 0.48		
	Hebron University	85	3.05 ± 0.83	3.33 ± 0.69		
	Palestine Polytech. Univ. (PPU)	65	3.10 ± 0.86	3.20 ± 0.67		
**Checkpoint Crossings**	Never	16	2.89 ± 0.79	3.13 ± 0.67	0.0046	0.729
	Rarely	39	2.96 ± 0.90	3.09 ± 0.74		
	Some rotations	175	3.31 ± 0.80	3.22 ± 0.63		
	Nearly all rotations	141	3.41 ± 0.84	3.22 ± 0.76		

In contrast, there were striking differences by university: students at Al-Quds University reported the highest burnout (3.55 ± 0.84) and lowest training adequacy (2.99 ± 0.72), whereas those at AAUP reported the lowest burnout (3.15 ± 0.79) and highest training adequacy (3.42 ± 0.48) (ANOVA Burnout: F(4, 366) = 6.12, *p* = 0.00009; Training: F(4, 366) = 4.55, *p* = 0.0013). Post-hoc contrasts indicated burnout at Al-Quds was significantly higher than at Hebron or PPU, and training adequacy at AAUP was higher than Al-Quds or PPU (all *p* < 0.01) (Table 2).

**Fig. 1:**
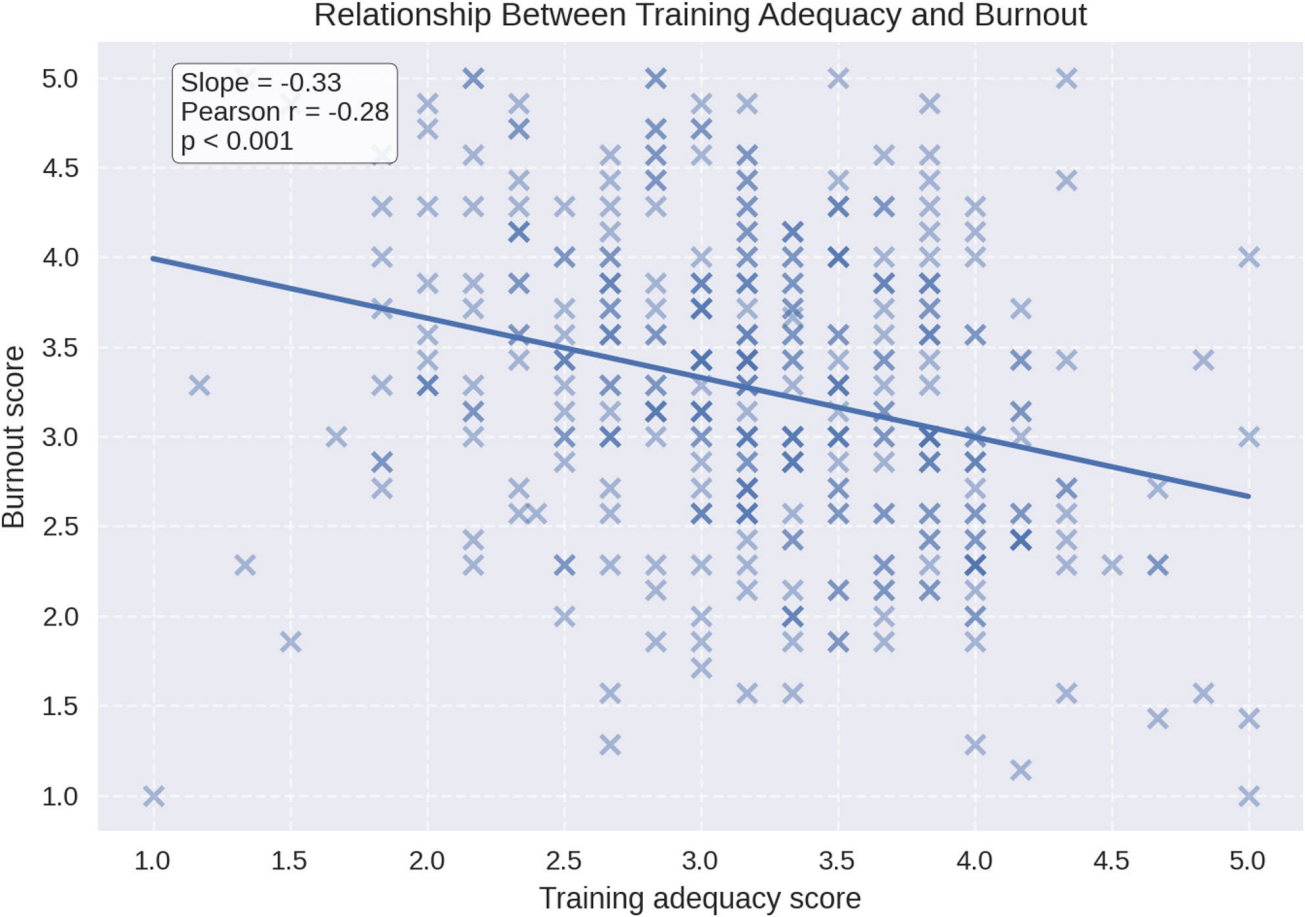
Relationship between perceived clinical training adequacy and burnout

Across primary outcomes, burnout and perceived training adequacy were negatively correlated (Pearson *r*=−0.276, *p*<0.0001), indicating that students with higher burnout tended to report lower perceived training adequacy (Figure 1). By contrast, perceived training adequacy was not associated with checkpoint burden (*r*=+0.022, p=0.668), whereas burnout increased modestly with checkpoint burden (*r*=+0.245, *p*<0.00001).

When dichotomizing checkpoint burden at the median, students with high burden (> median) had significantly higher burnout (3.38 ± 0.83) than those with low burden (3.20 ± 0.84; t(369) = 3.36, *p *= 0.001). Conversely, training adequacy did not differ by burden group (3.20 ± 0.69 vs. 3.19 ± 0.71; *p* = 0.89). In unadjusted comparisons, frequent checkpoint crossing and related delays were associated with worse outcomes. For example, students who crossed military checkpoints on “nearly all” rotations reported higher burnout (mean ± SD: 3.41 ± 0.84) than those who never crossed (2.89 ± 0.79); post-hoc t-tests showed that both 0 vs. 3 and 1 vs. 3 crossing-frequency comparisons were significant (*p* = 0.019 and *p* = 0.0038, respectively). Likewise, more frequent tardiness was linked to higher burnout (ANOVA F(4, 366) = 4.63, *p* = 0.0012); those who were “almost daily” late/missed parts of days had higher burnout (3.55 ± 0.89) than those who never were (2.95 ± 0.78). Likewise, students who had ever missed a full day of training due to checkpoint/roadblock issues had higher burnout than those who had not (one-way ANOVA F(2, 368) = 5.71, *p* = 0.0036).

Checkpoint-crossing frequency was common: 16 students (4.3%) reported never crossing military checkpoints, 39 (10.5%) rarely, 175 (47.2%) during some rotations, and 141 (38.0%) during nearly all or all rotations (Table 1). Mean burnout increased across these categories, from 2.89 ± 0.79 among students who never crossed checkpoints to 3.41 ± 0.84 among those who crossed during nearly all rotations (overall p=0.0046; Table 2).

We next examined perceived clinical training adequacy. Overall adequacy was modest (mean 3.18 ± 0.70). Although differences by demographic factors were smaller than for burnout, we found that training scores varied by university (Table 2) and were lowest among Al-Quds students (3.00 ± 0.72) and highest at AAUP (3.42 ± 0.48) (F(4, 366) = 4.55, *p* = 0.0013). No significant differences in training scores were seen by region (*p* = 0.121) or by checkpoint burden (*p* = 0.729).

Intention to pursue residency abroad was common (59.3% Yes). This outcome varied significantly by several factors (Table 3). Men were more likely than women to report intending to leave (67.3% vs. 52.3% saying “Yes”, χ² = 7.24, *p* = 0.007). Region of residence was strongly associated: 73.0% of students from the North West Bank (Jenin/Nablus/Tulkarm) intended to train abroad, versus only 35.3% of East Jerusalem students (χ² = 20.13, *p* = 0.00047). University also showed differences (χ² = 15.05, *p* = 0.0046): for instance, 75.6% of An-Najah students intended to leave compared to 48.7% at Al-Quds. Age groups did not differ significantly in overall intention (*p *= 0.096). Checkpoint burden group (above vs. below median) was not significantly associated with intent (*p *= 0.445). Notably, students who reported that checkpoint-related delays had reduced their clinical focus (“travel stress reduced my focus”) were more likely to intend to train abroad (endorsed by 85% of “Yes” vs. 74% of “No”, χ² = 5.12, *p *= 0.024).

**Table 3:** Intention to pursue residency training abroad by student characteristics. Values are counts and row percentages. χ² and *p* refer to tests of independence across all categories of each factor

Factor	Category	Intend Abroad: Yes *n* (%)	No/Unsure *n* (%)	χ²	*p*
**Gender**	Female	109 (52.3)	97 (47.7)	7.24	0.007
	Male	111 (67.3)	54 (32.7)		
**Age**	≤ 20	8 (66.7)	4 (33.3)	6.35	0.096
	21–22	121 (56.1)	92 (43.9)		
	23–24	83 (60.3)	55 (39.7)		
	≥ 25	8 (100.0)	0 (0.0)		
**Region**	South West Bank (Bethlehem/H.)	109 (60.9)	70 (39.1)	20.13	0.00047
	North West Bank (Jenin/Nablus)	65 (73.0)	24 (27.0)		
	East Jerusalem	18 (35.3)	33 (64.7)		
	Central West Bank (Ramallah)	19 (55.9)	15 (44.1)		
	1948 Territories	9 (50.0)	9 (50.0)		
**University**	Al-Quds Univ.	56 (48.7)	59 (51.3)	15.05	0.0046
	An-Najah Univ.	62 (75.6)	20 (24.4)		
	AAUP	13 (59.1)	9 (40.9)		
	Hebron Univ.	48 (54.5)	38 (45.5)		
	PPU	41 (62.1)	25 (37.9)		
**Checkpoint burden**	Low (≤ median)	124 (66.3)	63 (33.7)	0.58	0.445
	High (> median)	96 (52.2)	88 (47.8)		

Multivariable logistic regression (Table 4) was performed to identify independent predictors of intending to train abroad (Yes vs. No/Unsure). Higher burnout score was independently associated with greater odds of intending to train abroad (B = 0.451, SE = 0.142; adjusted OR 1.57, 95% CI 1.19–2.08; *p* = 0.0015). In contrast, higher checkpoint burden was associated with lower odds of intending to train abroad (B = -0.386, SE = 0.177; adjusted OR 0.68, 95% CI 0.48–0.96; *p *= 0.029). Male sex was also associated with higher odds (B = 0.779, SE = 0.230; adjusted OR 2.18, 95% CI 1.39–3.42; *p* = 0.0007), whereas residence in East Jerusalem was associated with lower odds (B = -1.347, SE = 0.338; adjusted OR 0.26, 95% CI 0.13–0.49; *p* < 0.0001). The model showed modest explanatory power (Nagelkerke pseudo-R² = 0.072).

**Table 4:** Multivariable logistic regression for intention to train abroad (Yes vs. No/Unsure)

Predictor	B (SE)	Adjusted OR (95% CI)	*p*-value
Checkpoint burden (per 1 SD increase)	-0.386 (0.177)	0.68 (0.48–0.96)	0.029
Burnout score (per 1-point increase)	0.451 (0.142)	1.57 (1.19–2.08)	0.0015
Male sex (ref = female)	0.779 (0.230)	2.18 (1.39–3.42)	0.0007
Residence in East Jerusalem (ref = other regions)	-1.347 (0.338)	0.26 (0.13–0.49)	< 0.0001

Outcome = intention to train abroad (Yes vs. No/Unsure). Predictors included checkpoint burden (standardized; OR per 1 SD increase), burnout score (OR per 1-point increase), sex (male vs. female), and residence in East Jerusalem (vs. other regions). Model pseudo-R² (Nagelkerke) = 0.072. B = logistic regression coefficient; SE = standard error; OR = odds ratio; CI = confidence interval

## Discussion

In this multi-centre cross-sectional study of Palestinian medical students, we found overall moderate levels of burnout and perceived training adequacy, alongside a strikingly high intention to seek residency training abroad. Burnout scores were higher among students exposed to a heavier checkpoint burden (more frequent crossings, longer delays, and repeated tardiness or missed days), among those studying at certain universities, and among residents of East Jerusalem and the 1948 territories. In contrast, perceived training adequacy varied mainly by university and was not significantly associated with checkpoint burden. In multivariable analysis, higher burnout and male gender independently predicted intention to train abroad, whereas living in East Jerusalem and higher checkpoint burden were associated with lower odds of expressing such intentions. Taken together, these findings suggest that “classical” educational stressors interact with daily structural violence checkpoints, movement restrictions, and fragmented health and educational systems to shape both well-being and early career trajectories.

Our results are broadly consistent with international estimates showing that approximately one-third to one-half of medical students worldwide meet criteria for burnout [16–18]. A meta-analysis of 50 studies estimated global burnout prevalence among medical students at about 37%, with higher rates in the Middle East than other regions [16]. Another meta-analysis reported pooled burnout prevalence of 44%, with particularly high levels of emotional exhaustion and depersonalization and no consistent gender difference [17]. A systematic review similarly linked burnout to depressive symptoms, suicidal ideation, and thoughts of dropping out of medical school [18]. Within the region, studies from Iraq report burnout in nearly 40% of medical students, with very high levels of emotional exhaustion and cynicism [19]. Our mean burnout scores fall within this international range, reinforcing that Palestinian students share the global burden of burnout, but experience it within an especially adverse socio-political context.

The Palestinian context is distinguished by chronic occupation, military checkpoints, and recurrent exposure to violence, all of which have been shown to undermine mental health and quality of life in the general population [20]. A cross-sectional study from the Gaza Strip reported extremely high levels of depression, anxiety, stress, poor sleep, and life dissatisfaction among medical students, clearly exceeding global pooled estimates [21]. Other work from Palestine has demonstrated strong associations between reduced quality of life and higher levels of depression, anxiety, and stress in adults living under ongoing political violence [20]. Among Palestinian nursing students, exposure to violence at checkpoints was almost universal, with particularly poor scores in environmental and psychological quality-of-life domains during the current war [22]. In this context, our finding that greater checkpoint burden is associated with higher burnout is consistent with broader evidence linking structural oppression and mobility restrictions to psychological distress and impaired functioning among Palestinians [20, 22–25].

The checkpoint burden index we constructed integrating frequency of crossings, delay duration, and consequent tardiness or absence captures a dimension of chronic, externally imposed stress that is rarely quantified in medical education research. Geographic and health-systems analyses have documented how checkpoints, roadblocks, and the separation wall fragment Palestinian territories and restrict access to hospitals and clinics [24, 25]. These barriers increase travel time, unpredictability, and exposure to harassment and violence, especially for students commuting to urban academic centres. Our findings extend this literature by showing that such structural constraints are not only barriers to care and service delivery but also significant determinants of burnout among future physicians. Notably, checkpoint burden was not significantly associated with perceived training adequacy, suggesting that clinical teaching and supervision may be maintained despite disruptions, but at a substantial psychological cost to students.

Burnout and training adequacy also varied significantly by university and region. Students at institutions requiring long commutes through multiple checkpoints reported higher burnout and, in some cases, lower training adequacy scores. These differences mirror patterns seen in other conflict-affected settings, where educational quality and trainee well-being differ sharply between institutions depending on local infrastructure, security conditions, and availability of faculty [26, 27]. In Syria, for example, protracted conflict has produced profound disruptions in clinical training, faculty migration, and resource allocation across regions and medical schools [26, 27]. Our findings suggest similar intra-national inequities in Palestine, where students based in the central West Bank and those commuting to East Jerusalem hospitals may experience disproportionate stress due to compounded academic and political pressures.

The exceptionally high proportion of students expressing a desire to pursue residency abroad underscores the risk of further “brain drain” from an already fragile health system. Intentions to emigrate among medical and other health-profession students are driven by complex combinations of economic, professional, and security concerns [28, 29]. A systematic review of health-worker migration from low- and middle-income countries identified low remuneration, poor working conditions, limited career prospects, and insecurity as key push factors [28]. A scoping review of medical students’ international migration similarly highlighted dissatisfaction with local training, perceived lack of meritocracy, and political instability as major drivers [29]. In our study, higher burnout was independently associated with wanting to train abroad, aligning with evidence that burnout is linked to career regret and intentions to abandon training or practice in the local system [17, 18, 30]. For Palestinian students, burnout may function both as a marker of personal distress and as a proxy for perceptions that local systems cannot provide safe, rewarding, and sustainable career paths.

The inverse association between checkpoint burden and intention to train abroad is counter-intuitive and warrants careful interpretation. One possible explanation is that students who experience the heaviest restrictions often those with West Bank IDs needing special permits may perceive international training as logistically unattainable, regardless of distress. By contrast, students with greater mobility (e.g., Jerusalem or 1948 IDs, foreign passports, or stronger financial resources) may more readily translate dissatisfaction and burnout into concrete emigration plans. This interpretation is consistent with broader literature suggesting that international migration is often more feasible for individuals with higher socioeconomic capital or fewer mobility constraints, even when distress is widespread [28, 29]. It also resonates with qualitative accounts of Palestinian health workers who describe feeling “trapped” by bureaucratic and political barriers despite high motivation to leave unsafe or under-resourced workplaces [23, 24].

Gender differences in emigration intentions also emerged, with male students more likely to report intention to seek training abroad even after adjusting for burnout and other covariates. Global evidence on gender and health-worker migration is mixed, but several studies suggest that men are more likely to undertake long-distance migration for postgraduate training or work, whereas women may face greater family responsibilities, social expectations, or safety concerns that restrict mobility [28, 29]. In patriarchal and conflict-affected settings, these constraints may be amplified, with women disproportionately bearing caregiving and household stability burdens during crises.

The broader literature on healthcare worker burnout in Palestine and the wider region reinforces the urgency of addressing these issues early in the training pipeline. Recent studies report high burnout levels among critical-care workers, anesthesia providers, and frontline staff in Palestinian hospitals, with political instability and economic hardship frequently cited as major contributors [23, 26]. During the ongoing war in Gaza, burnout, anxiety, depression, and moral injury among healthcare workers have reached extreme levels, reflecting workplace danger, resource scarcity, and repeated exposure to mass casualty events [21, 23, 26]. Systematic reviews focusing on the Middle East, North Africa, and Turkey have confirmed high pooled burnout prevalence in healthcare workers and identified heavy workloads, low autonomy, and exposure to violence as key risk factors [23]. Similar patterns have been observed across conflict-affected health systems globally, including in Syria and neighbouring countries, where attacks on health facilities, displacement of health workers, and chronic insecurity undermine both service delivery and training [26, 27].

The implications for policy and practice are substantial. At the institutional level, medical schools in Palestine can adopt evidence-informed strategies shown to mitigate burnout in trainees, including longitudinal mentorship programmes, protected time for self-care, peer-support groups, and structured debriefings after particularly stressful clinical exposures [16–18]. Curricular reforms that reduce unnecessary workload, improve clarity of expectations, and foster a supportive learning environment have been associated with lower burnout in other settings [16, 17]. However, individual-level and curricular interventions alone are unlikely to be sufficient when students’ daily commutes involve unpredictable delays, exposure to violence, and constant uncertainty about access. Meaningful improvement will also require structural changes such as secure dormitory options near clinical sites, flexible rotation scheduling that anticipates checkpoint delays, and advocacy with relevant actors to protect the right to education and healthcare under occupation [22–25, 31].

This study has several strengths. To our knowledge, it is one of the first multi-institutional assessments of burnout, perceived clinical training adequacy, checkpoint burden, and intention to train abroad among medical students in Palestine. We examined both educational and structural predictors and used context-adapted composite measures, including a novel checkpoint-burden index. Several limitations should be acknowledged. First, the cross-sectional design precludes causal inference. Second, all measures were self-reported and may be affected by recall and social-desirability bias. Third, the burnout and training-adequacy scales were adapted for this study rather than administered as previously validated instruments in their original form; although internal consistency was acceptable, we did not perform full psychometric validation, including factor analysis and broader evaluation of construct validity and scoring assumptions. These scores should therefore be interpreted as exploratory composite measures. Fourth, convenience and snowball sampling may have introduced selection bias and may limit generalizability. Finally, our checkpoint-burden index does not capture all dimensions of structural violence, such as raids, settler violence, or university closures, that may also influence student well-being and career intentions.

Future research should build on these findings using longitudinal designs to track burnout, mental health, and career intentions over time and across phases of conflict. Mixed-methods studies could illuminate how students interpret and cope with daily movement restrictions, academic pressures, and political violence, and how these experiences influence professional identity and social responsibility. Intervention studies evaluating mentorship programmes, peer-support groups, or institutional policy changes are urgently needed. At the same time, international bodies, academic partners, and policymakers must recognise that burnout among Palestinian medical students is not merely an individual or institutional problem; it is deeply rooted in structural conditions that impede freedom of movement, undermine access to education and healthcare, and systematically erode the human resources on which any future health system will depend [23–25, 28, 29].

## Conclusion

This multi-centre study shows that Palestinian medical students experience common burnout, pervasive movement restrictions, and high intentions to pursue residency abroad. Burnout was associated with checkpoint burden and independently predicted plans to train overseas, yet those with the greatest mobility constraints were less likely to report intentions to leave, suggesting that emigration is shaped by opportunity as well as need. Although perceived training adequacy appears preserved, these conditions may accelerate “brain drain” and weaken the future specialist workforce. Coordinated institutional, national, and international action is needed to reduce burnout, strengthen local training pathways, and address structural barriers that drive distress and constrain mobility.

## Data Availability

The datasets used and/or analyzed during the current study are available from the corresponding author on reasonable request.
